# Genome-Wide Analysis of the Auxin/Indoleacetic Acid Gene Family and Response to Indole-3-Acetic Acid Stress in Tartary Buckwheat (*Fagopyrum tataricum*)

**DOI:** 10.1155/2021/3102399

**Published:** 2021-10-26

**Authors:** Fan Yang, Xiuxia Zhang, Ruifeng Tian, Liwei Zhu, Fang Liu, Qingfu Chen, Xuanjie Shi, Dongao Huo

**Affiliations:** ^1^Henan Academy of Agricultural Sciences, Zhengzhou 450002, China; ^2^Guizhou Normal University, Guiyang 550025, China; ^3^College of Plant Science & Technology, Huazhong Agricultural University, Wuhan 430070, China; ^4^Zhengzhou University, Zhengzhou 450001, China

## Abstract

Auxin/indoleacetic acid (Aux/IAA) family genes respond to the hormone auxin, which have been implicated in the regulation of multiple biological processes. In this study, all 25 Aux/IAA family genes were identified in Tartary buckwheat (*Fagopyrum tataricum*) by a reiterative database search and manual annotation. Our study provided comprehensive information of Aux/IAA family genes in buckwheat, including gene structures, chromosome locations, phylogenetic relationships, and expression patterns. Aux/IAA family genes were nonuniformly distributed in the buckwheat chromosomes and divided into seven groups by phylogenetic analysis. Aux/IAA family genes maintained a certain correlation and a certain species-specificity through evolutionary analysis with *Arabidopsis* and other grain crops. In addition, all Aux/IAA genes showed a complex response pattern under treatment of indole-3-acetic acid (IAA). These results provide valuable reference information for dissecting function and molecular mechanism of Aux/IAA family genes in buckwheat.

## 1. Introduction

Tartary buckwheat (Fagopyrum tataricum), also named as bitter buckwheat or kuqiao, is an annual eudicot plant belonging to the genus Fagopyrum [1]. It is originated in southwest China and currently grown on western China, Japan, South Korea, Canada, and Europe, for exhibits strong abiotic resistance to harsh eco-climatic environments [2, 3]. Buckwheat is considered an important medicinal and edible food crop, rich in protein, and a balance of essential amino acids, as well as beneficial phytochemicals. ([4–6]. Flavonoids, especially rutin, significantly higher than in other crops, have antifatigue properties and anti-inflammatory activity and can be used to treat microangiopathy [7]. The study on the mechanism of the important metabolites can effectively promote the use of buckwheat. In addition, studying the resistance mechanism of buckwheat is not only beneficial to the production of buckwheat under stress but also can get meaningful resistance genes for other crops. Auxin plays an important role in controlling multitudinous vital processes [8–11] and stress tolerance ([12–14]. It is significant to study the response of buckwheat to hormones.

The classical plant hormones, including auxins, cytokinins, gibberellins, abscisic acid, and ethylene, were discovered several decades ago. Recently, a number of additional molecules have been identified that might also be classified as plant hormones. While a considerable amount is known about the biosynthesis and distribution of these hormones in plants, the receptors and signal transduction pathways of plant hormones are only beginning to be unraveled. Auxin has many roles in plant growth and development. It mediates elongation of stem and root growth, enlargement of fruits and tubers, and promotion of cell division, through regulating cell division, expansion, differentiation, and patterning [15, 16]. In an attempt to understand the molecular mechanism of auxin action, six gene families that regulating auxin-responsive have been identified and characterized from different species, which including the auxin response factor (ARF) gene family [17], small auxin-up RNA (SAUR) gene family [18–20], Gretchenhagen-3 (GH3) gene family [21, 22], Auxin input carrier (AUX1) gene family [23], Transport inhibitor response 1 (TIR1) gene family [24], and auxin/indoleacetic acid (Aux/IAA) gene family [25, 26].

Dynamic spatial and temporal changes in auxin levels can trigger gene reprogramming precisely and rapidly, which requires auxin early response genes, such as the Aux/IAA, ARF, SAUR, and GH3 families. Among these genes, Auxin/indole-3-acetic acid (Aux/IAA) family numbers have identified as short-lived nuclear proteins that represent a class of primary auxin-responsive genes and play a pivotal role to perception and signaling of the plant hormone auxin [27, 28]. At high auxin levels, Aux/IAA proteins can be ubiquitinated by interacting with TIR1/AFB receptors and subsequently degraded via the 26S proteasome [29, 30], the different protein results in distinct auxin-sensing effects in different tissues and developmental phases [31, 32], thereby regulating the processes of plant growth and development in a precise manner.

The first isolated Aux/IAA genes were the PS-IAA4/5 and PS-IAA6 genes from pea [33, 34]. Subsequently, 14 Aux/IAA genes were isolated from Arabidopsis based on the homologues to the genes from pea [35]. With the advent of genome sequencing, the IAA/Aux gene family has been identified in more than 30 plant species by genome-wide analysis ([36–39]. Over the past two decades, members of this family have been intensely studied in Arabidopsis and shown to have distinct functions in plant growth and development processes. The mechanism by which the Aux/IAA gene family responds to auxin stimulation has been effectively analyzed [40]. Aux/IAA genes encode short-lived nuclear proteins, comprising four highly conserved domains [41], namely, domains I and II, which are located at the N-terminus, and domains III and IV located at the C-terminus. Domain I has the amphiphilic motif LXLXLX that is associated with ethylene response factors, can bind to corepressors, and is required for the transcriptional inhibitory function of Aux/IAA proteins [40, 42]. The domain II core sequence VGWPP is the target of Aux/IAA protein ubiquitination for degradation [43–45]. Domains III and IV are sites that bind to the auxin response factor, and their secondary structure can be folded into a helix-roentle-helix motif. Domain IV may also contribute to the dimerization. Furthermore, in domains II and IV, there are generally two nuclear localization signals (NLS) [46]. In addition, the phosphorylation site of photosensitive pigments between domains I and II suggests that the Aux/IAA protein could mediate the auxin and optical signaling pathways through phosphorylation of the photosensitive pigments [47]. While considerable information has been obtained about the biosynthesis and distribution of these hormones in plants, the receptors and signal transduction pathways for plant hormones are only beginning to be unraveled.

Sequences derived from large-scale sequencing projects are informative in functional genomics research, providing an opportunity to scan gene families. Since the first publication of the buckwheat genome sequence, understanding of the genome information of buckwheat has been greatly enhanced [3]. In this study, we identified at least 25 putative members of buckwheat Aux/IAA genes using a special Aux/IAA domain hidden Markov model (HMM) of the whole genome. Therefore, we performed bioinformatics analyses, including phylogenetic, gene structure, and motif composition analyses, to determine the chromosomal locations of the genes. Subsequently, phylogenetic comparisons with Arabidopsis and other crops were performed. This study contributes to the clarification of the functions of Aux/IAA proteins and provides a foundation for further comparative genomic studies in Tartary buckwheat.

## 2. Results

### 2.1. Identification and Annotation of the Aux/IAA Genes in Tartary Buckwheat

A total of 25 genes (shown in Table 1) were identified using Basic Local Alignment Search Tool (BLAST) methods through the conserved sequences generated from the HMM profile in Pfam using the 261 aa conserved sequences of Aux/IAA proteins based on the potential orthologs in Arabidopsis. The genes confirmed to contain conserved domains of Aux/IAA proteins, and the transcripts with the lowest *E*-value of domain examination were named FtAux/IAA genes. Gene sequence analysis of the 25 FtAux/IAAs showed that the predicted protein lengths were 160 and 890 aa, and the CDS sequences varied in size from 540 bp to 2673 bp. Moreover, the pI (theoretical isoelectric point) and MW (molecular weight) ranged from 5.4 to 9.15 and 20280.1 kDa to 99377.01 kDa, respectively.

### 2.2. Chromosomal Locations of FtAux/IAA

**Figure 1:**
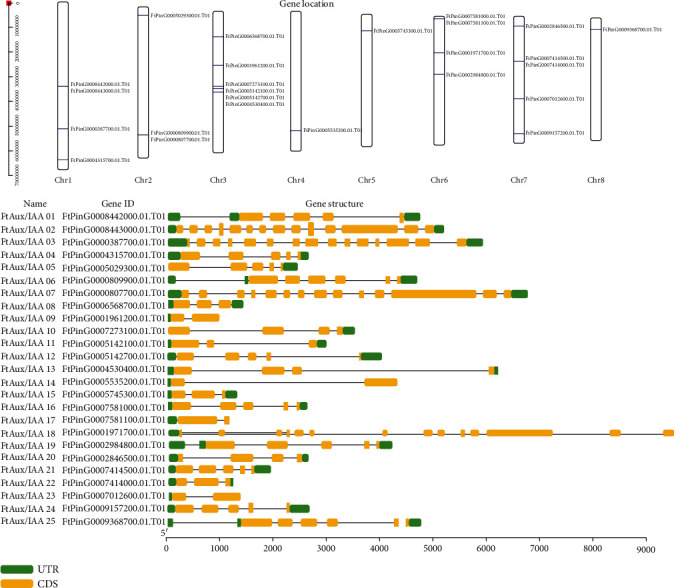
Distribution and gene structure of *FtAux/IAA* genes among eight chromosomes. Constrictions on the chromosomes (vertical bar) indicate the position of genes. The chromosome numbers and sizes (Mb) are indicated at the top of each bar. The UTR and exon-intron organization of the *FtAux/IAA* genes. The UTRs and exons and introns are represented by boxes and lines, respectively.

The FtAux/IAA gene sequences were initially mapped onto the Tartary buckwheat genome, and all 25 FtAux/IAA genes were separately mapped onto eight chromosomes. Most FtAux/IAA genes were observed at the top and bottom arms of the chromosomes, and a cluster was distributed on different chromosomes (Figure 1). Four genes (16%) were located on Chr. 1, and three genes on Chr. 2, which comprised 12% of the total number of genes. Chr. 3 had six FtAux/IAA genes, which was the highest number in a single chromosome. The lowest proportion of genes (4%) was on the Chr. 4, Chr. 5, and Chr. 8, containing one gene each. There were four (15%) and five (20%) genes on Chr. 6 and Chr. 7, respectively. In terms of distribution, the genes of different families remained relatively regional, with all but a few of the 31 genes in the cluster, whose number was between two and three decibels. In addition, the genes FtAux/IAA 01 and FtAux/IAA 02 were located adjacent to each other on the first chromosome and showed a tight chain. The same observation was found on Chr. 2, Chr. 3, Chr. 6, and Chr. 7, where there were two, four, two, and two closely linked genes, respectively. These data suggest that the distribution of some FtAux/IAA genes on the buckwheat genome probably results from either reverse or direct tandem duplication.

The genes in the same evolutionary group have a similar structure and tend to have similar gene functions, which as it has been shown in other species, such as Arabidopsis and rice [48]. We analyzed the structure of introns and exons of the FtAux/IAA gene sequences using the plaza database (https://bioinformatics.psb.ugent.be/plaza/versions/plaza) of full-length cDNA (Figure 1). All FtAux/IAAs had different numbers of exons and introns in the translated region; the number of introns and exons varied from 1 to 14 and 2 to 15, respectively. Four genes (FtAux/IAA9, FtAux/IAA14, FtAux/IAA17, and FtAux/IAA23) contained two exons and one intron. FtAux/IAA8, FtAux/IAA15, and FtAux/IAA22 contained three exons and two introns. Genes with four exons were FtAux/IAA10, FtAux/IAA13, and FtAux/IAA20. There were eight genes, namely, FtAux/IAA1, FtAux/IAA4, FtAux/IAA5, FtAux/IAA12, FtAux/IAA16, FtAux/IAA19, FtAux/IAA21, and FtAux/IAA24, with five exons. FtAux/IAA6 and FtAux/IAA25 had six exons and five introns. There were three genes (FtAux/IAA2, FtAux/IAA7, and FtAux/IAA18) containing 14 exons, and FtAux/IAA3 contained the most number of exons. In general, genes of the FtAux/IAA family showed rich structural variation in buckwheat and may be involved in various metabolic regulatory networks and developmental processes.

### 2.3. Gene Peptide Sequence and Motif Composition of the FtAux/IAA Gene Family

**Figure 2:**
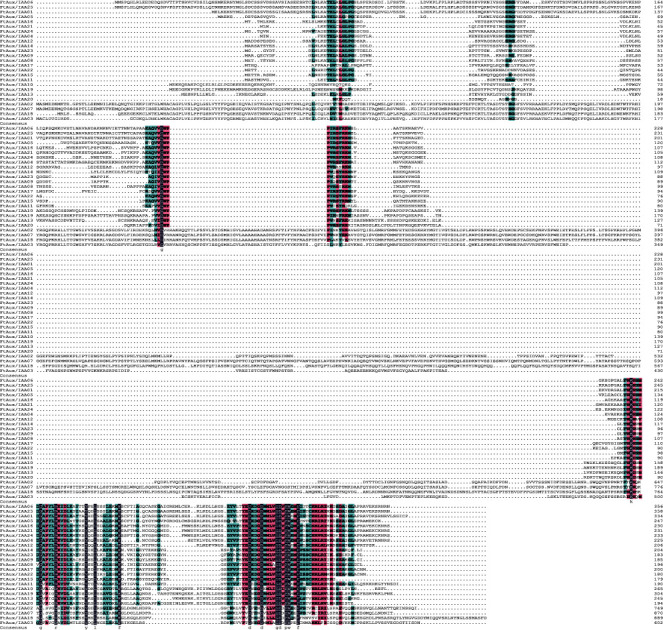
Multiple sequence alignment of the full-length FtAux/IAA proteins obtained with DNAMAN. Conserved domains of FtAux/IAA proteins are underlined. The gene ID is mentioned on the left of each sequence and amino acid position on the right of each sequence.

The peptide sequences of all 25 FtAux/IAAs are shown in Figure 2; all the results were verified using DNAMAN. The overall identity of the various proteins is low, which is similar to those of the Aux/IAA polypeptides previously determined in other plants. To examine in detail the domain organization of FtAux/IAA proteins, multiple sequence alignments of the full-length protein sequences were performed using the ClustalX program. Alignment of the amino acid sequences of FtAux/IAA revealed four typical highly conserved domains [34]. According to the Pfam outcome of the protein sequences, most of the genes contained four conserved structures, except for the missing domain I in the genes FtAux/IAA10 and FtAux/IAA17. In the second domain, many of the variations were the same in domains II, III, and IV. A pairwise analysis of the full-length FtAux/IAA protein sequences indicated that the overall identities ranged from 19% to 69%. However, the amino acid identity within the conserved domains reached 90%. Domain I contained a leucine-rich region and was the least conserved among the family members. The proline-rich domain II was comparatively more conserved. The classification of all the genes as Aux/IAA family members was confirmed by constructing a phylogenetic tree based on domain III and IV amino acid sequences of the 25 FtAux/IAA and two representative proteins. Amino acid sequence analysis yielded the same results as the gene structure analysis and the same results as other gene family analyses.

### 2.4. Gene Structure and Motif Composition of the FtAux/IAA Gene Family

**Figure 3:**
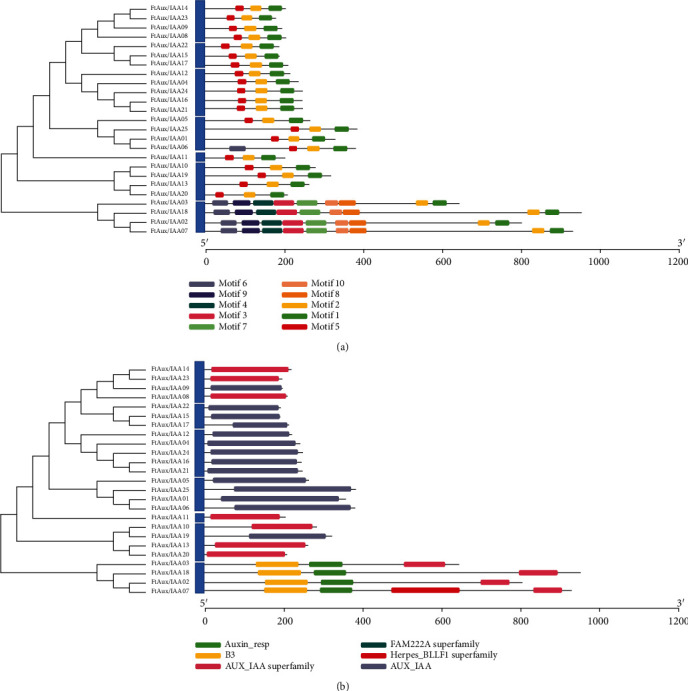
Gene motif pattern and gene domains in *FtAux/IAA* genes from Tartary buckwheat. (a) The protein domains of *FtAux/IAAs* are shown and are denoted by rectangles with different colors. (b) Gene domains with different functions are shown in different colored boxes.

To study the evolutionary relationship of the buckwheat Aux/IAA family gene, a phylogenetic tree was constructed using the amino acid sequences of the FtAux/IAA genes. The sequences of buckwheat Aux/IAA proteins were further analyzed using the online software MEME to understand the diversity and evolutionary relationships.  Figure 3 shows that FtAux/IAA proteins are grouped into seven distinct clades, and each group contains a different number, between one and five, of members of the FtAux/IAA family. In group I, there were four members of FtAux/IAA14, FtAux/IAA23, FtAux/IAA09, and FtAux/IAA08. Group II contained three members: FtAux/IAA22, FtAux/IAA15, and FtAux/IAA17. The five most common genes were FtAux/IAA12, FtAux/IAA04, FtAux/IAA24, FtAux/IAA16, and FtAux/IAA21. In group IV, four members named FtAux/IAA05, FtAux/IAA25, FtAux/IAA01, and FtAux/IAA06 were on the branch. In group V, there was only one gene, FtAux/IAA11. FtAux/IAA10, FtAux/IAA19, FtAux/IAA13, and FtAux/IAA20 comprised group VI, and four genes (FtAux/IAA03, FtAux/IAA18, FtAux/IAA02, and FtAux/IAA07) comprised group VII. In all groups, seven sister gene pairs were found to have a relatively close relationship with other FtAux/IAA family members in the evolutionary tree. These results indicate that the functions of the FtAux/IAA genes in different groups are diverse.

The motifs with similar functional domain distributions were highly conserved in family genes, although there were significant differences (Figure 3(a)). In general, these genes can be divided into two categories, with 20 genes carrying three identical motifs and the gene FtAux/IAA containing four motifs other than motif six, in addition to the same sequence of motif 5-2-1. In addition, FtAux/IAA02, FtAux/IAA03, FtAux/IAA07, and FtAux/IAA18 contained nine motifs, 6-9-4-3-7-10-8-2-1. These results are similar to those reported in a previous study, suggesting that these motifs may contribute to the specific functions of these genes [49]. Gene domains with different functions are shown in Figure 3(b), with 14 genes containing only the Aux/IAA domain and seven genes containing only the herpes BLLF 1 superfamily domain and all the genes belonging in groups I to VI. The FtAux/IAA03, FtAux/IAA18, and FtAux/IAA02 genes in group VII had three domains: B3, Auxin-resp, and Aux/IAA superfamily; gene FtAux/IAA07 had four domains in the order B3, Auxin-resp, herpes BLLF 1 superfamily, and Aux/IAA superfamily.

### 2.5. Phylogenetic Analysis of the FtAux/IAA Genes in Maize, Arabidopsis, Rice, and Sorghum

**Figure 4:**
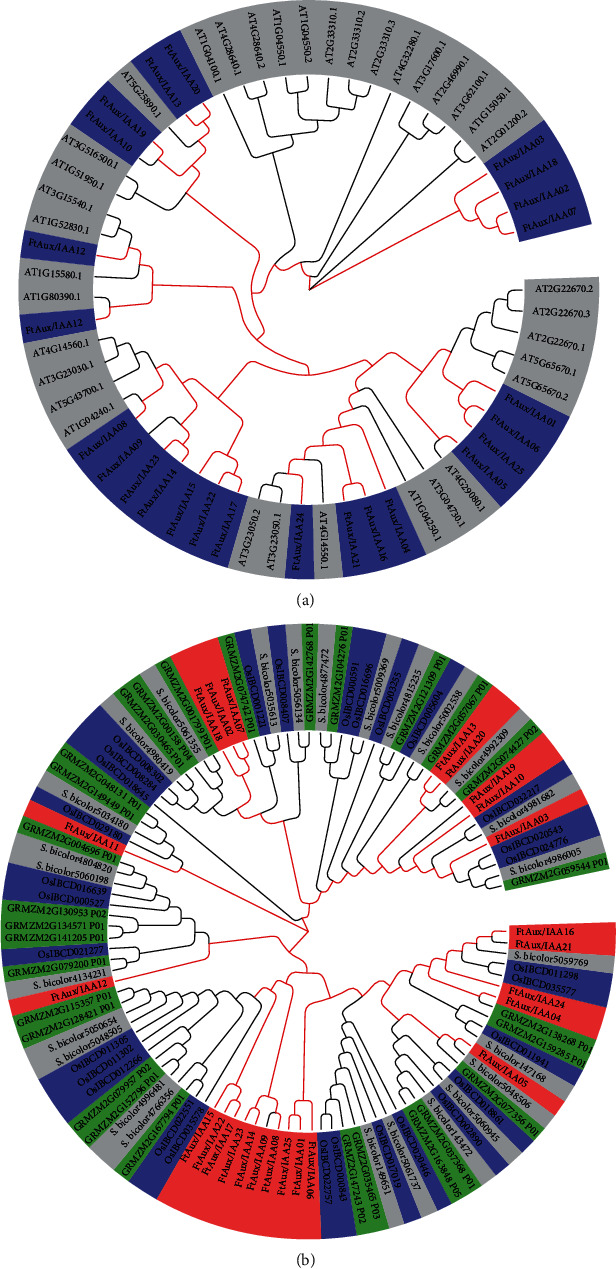
Phylogenetic relationship of Aux/IAA proteins. (a) The tree was reconstructed using Aux/IAA sequences of *Arabidopsis thaliana* (gray) and buckwheat (blue). Evolutionary distances were computed using the p-distance method and expressed in units of the number of amino acid substitutions per site. (b) The tree was reconstructed using Aux/IAA sequences in *Oryza sativa* (blue), *Sorghum bicolor* (gray), *Zea mays* (green), and buckwheat (red). Evolutionary distances were computed using the p-distance method and expressed in units of the number of amino acid substitutions per site.

In order to analyze the phylogenetic organization, we performed a phylogenetic analysis of 25 buckwheat Aux/IAAs and 36 Arabidopsis Aux/IAAs by generating a phylogenetic tree based on the neighbor-joining (NJ) method using MEGA [50]. Based on their phylogenetic relationships, we divided these Aux/IAAs into 10 groups, designated as groups I to X (Figure 4(a)). The family genes showed stronger clustering between buckwheat and Arabidopsis, and the nodes at the base of the larger clades were not well supported, but the nodes at the base of many smaller clades were robust. Buckwheat genes were concentrated in groups I, VI, VII, VIII, IX, and X. Genes in group I were all buckwheat, and groups II, III, IV, and V contained only Arabidopsis genes. In the other groups, the genes were distributed in both buckwheat and Arabidopsis. Phylogenetic analysis was performed using 30 rice (blue), 28 maize (green), 26 sorghum (gray), and 25 buckwheat (red) genes. Interestingly, using phylogenetic analyses, some Aux/IAA genes were suggested to form species-specific clades or subclades after the divergence of these species in this study.

### 2.6. The Expression of Aux/IAA Gene Family in Tartary Buckwheat

**Figure 5:**
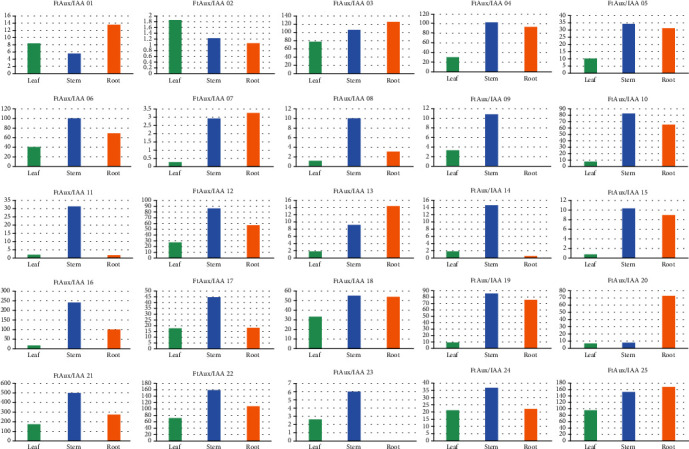
Expression of *FtAux/IAA* genes in different tissues from Tartary buckwheat. qRT-PCR was used to assess *FtAux/IAA* gene transcript levels in total RNA samples extracted from the leaves, stems, and roots of seeding plants at the two-leaf stage.

To examine the physiological roles of the FtAux/IAA genes and their response to auxin, we examined their expression in the roots, stems, and leaves at the two-leaf stage. The results of quantitative reverse transcription-polymerase chain reaction (qRT-PCR) showing the expression of FtAux/IAA family genes in Tartary buckwheat in different tissues are presented in Figure 5. Overall, all genes except FtAux/IAA09 and FtAux/IAA23 were expressed in all three tissues. In the leaves, the expression levels of different genes varied greatly, and the relative expression levels ranged from 1 to 3. Among all genes, FtAux/IAA02 had the highest expression levels. However, the expression levels of most genes in the leaves were significantly lower than those in the stem and root tissues. The expression levels of the genes FtAux/IAA01, FtAux/IAA03, FtAux/IAA07, FtAux/IAA13, FtAux/IAA18, FtAux/IAA20, and FtAux/IAA25 in stem tissue were lower than those in the leaf tissue, although different genes had higher expression levels in the stem tissue. FtAux/IAA09 and FtAux/IAA23 genes were not expressed, FtAux/IAA11 and FtAux/IAA13 genes were slightly expressed, and the remaining genes were primarily expressed in the roots. The tissue expression results showed that 18 genes expressed at high levels in the stem, and four genes, FtAux/IAA08, FtAux/IAA09, FtAux/IAA11, and FtAux/IAA14, had significantly higher levels of expression in the stem than in the leaf and root. FtAux/IAA02 had higher expression in the leaves than in the roots and stems. FtAux/IAA01, FtAux/IAA03, FtAux/IAA07, FtAux/IAA13, FtAux/IAA20, and FtAux/IAA25 genes had the highest expression in the roots. In addition, some genes showed significantly higher tissue expression than other member genes. The relatively high expression in different tissues suggests that the genes might play a role in seedling plant growth. Expression levels were always in the middle of the upper levels of different tissue expressions. These results are similar to those of previous functional studies on soybean [51] and Arabidopsis thaliana [52].

**Figure 6:**
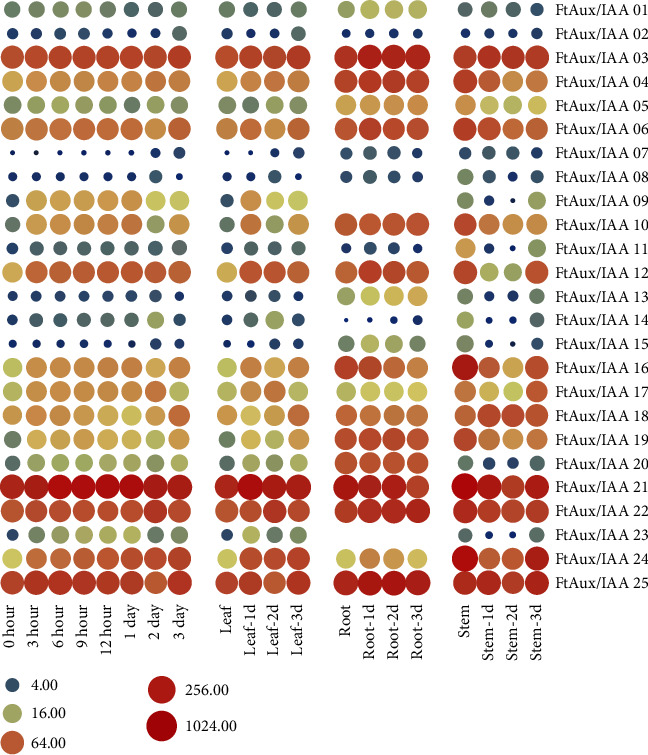
The pattern of transcript levels of 25 *FtAux/IAA* genes in buckwheat after IAA treatment compared with that of the control in different tissues. qRT-PCR was used to assess FtAux/IAA gene transcript levels in total RNA samples extracted from the leaves, stems, and roots after IAA treatment at the two-leaf stage.

As an important gene family that responds to auxin signaling, Aux/IAA is the most essential gene family that is regulated by exogenous IAA. The expression patterns of FtAux/IAAs in plantlets after IAA treatment were investigated using qRT-PCR. After treatment with 10 *μ*mol L^–1^ IAA for 3, 6, 9, and 12 h, expression of Aux/IAA genes was consistently upregulated compared to that of the control (Figure 6). The expression levels of all 25 FtAux/IAAs displayed a similar pattern in response to IAA treatment, and the expression levels were upregulated in all tissues. In addition, we found that the expression levels of FtAux/IAAs showed different degrees of increase under short-time IAA treatment, which is similar to the results of previous studies [53]. After IAA treatment for 1 day, 2 days, and 3 days, expression of genes showed diversity in trends, and the expression of genes such as FtAux/IAA04, FtAux/IAA07, FtAux/IAA14, and FtAux/IAA24 was significantly upregulated over time. However, expression of the genes FtAux/IAA01, FtAux/IAA02, FtAux/IAA06, FtAux/IAA10, FtAux/IAA12, FtAux/IAA16, FtAux/IAA17, FtAux/IAA21, and FtAux/IAA25 was first upregulated and then downregulated. None of these was downregulated upon long-term treatment. In general, different genes showed different trends upon treatment for longer periods of time. The expression of some genes was also different in the different tissues.

## 3. Discussion

Auxin signaling is a key signaling pathway in many plant biological processes, such as growth, organogenesis, and response to a variety of environmental changes [54–56]. Among the six auxin-related gene families (Aux/IAA, ARF, GH3, SUAR, AUX1, and TIR1), Aux/IAA is very important, representing a class of primary auxin-responsive genes, which are rapidly induced by auxin [57]. Therefore, studies on the function of the Aux/IAA gene family are beneficial for the analysis of plant development, stress resistance, and other biological processes, as a gene family directly responding to IAA treatment [52, 58, 59]. In recent years, a large number of Aux/IAA genes that regulate auxin signal transduction and auxin degradation have been identified in various plants ([25, 39, 52, 60] by the comprehensive application of physiological, genetic, molecular, and biochemical methods [15]. The complete genomic sequence has opened new avenues for understanding the plant genome and identifying the gene family [3] in Tartary buckwheat.

The comprehensive identification and subsequent characterization of the Tartary buckwheat Aux/IAA gene family members described here provide new insights into the potential role of some Aux/IAA genes in mediating plant responses to auxin, their putative function, and their mode of action. In this study, 25 FtAux/IAA genes were identified, and the number of FtAux/IAA members from Tartary buckwheat was found to be comparable to that of Arabidopsis [52, 61], rice [25], maize [39], tomato [36], cucumber [37], hybrid aspen [60], chickpea, and soybean [62, 63], although their genome sizes are quite different. These results indicate that the Aux/IAA gene family exists widely in the plant kingdom. Phylogenetic comparison of Aux/IAA proteins between Tartary buckwheat and Arabidopsis thaliana showed that there were genes similar to Arabidopsis thaliana genes in all but two branches. In addition, Tartary buckwheat had two independent branches, which had no corresponding Arabidopsis thaliana genes. The same trends were observed in the comparisons with rice, maize, sorghum, and other species. As an illustration of the wide diversification of Aux/IAA proteins in higher plants, the two clades are also expanded in Populus trichocarpa [38] and Solanum lycopersicum [36]. This diversification is also reflected by the important structural variations found within the Aux/IAA proteins. This partially accounts for the Aux/IAA conservation in these species during the evolutionary process ([25, 39, 64, 65]. Twelve of the 25 FtAux/IAA loci formed six sister pairs in the NJ reconstructions, four of which had strong bootstrap support, indicating that Aux/IAA genes in Tartary buckwheat may play nonredundant roles during plant development. Considering that their expression pattern is apparently restricted to narrow developmental stages and their atypical long-lived features, the buckwheat noncanonical Aux/IAA proteins may have a specific function in mediating auxin responses during well-defined plant developmental events.

Gene structure analysis showed that the genes of this family contained 2–15 exons and 1–14 introns. Eighteen of the genes had UTR regions at either ends of the genes, and another seven lacked UTRs at either ends. According to motif structure, family genes can be divided into two groups. One group had more than nine motif structures and showed consistent sequences; however, there were differences in location and gene length. In the other group, 21 genes showed 3–4 motifs. These conserved motifs comprised several major conserved structures in the Aux/IAA family, such as the Aux/IAA superfamily, Aux/IAA, and Herpes BLLF1 segments. These results show that a large proportion of Aux/IAA genes was produced by gene repeat events, such as segmental, tandem, or both, in the course of evolution [62, 66], and the expanded Aux/IAA gene members in land plants create functional redundancy and may be associated with new functions to adapt to environmental changes [63, 67, 68].

Gene expression patterns in Tartary buckwheat seedlings and responses to short- and long-term hormonal stimuli were identified using qRT-PCR analysis, providing new insights regarding the potential role in mediating plant responses to auxin. Transcript abundance in particular organs at a given time is an important prerequisite for the subsequent elucidation of the corresponding proteins required for proper execution of developmental, metabolic, and signaling processes. Virtually, all 25 FtAux/IAA genes were expressed in all organs/tissues analyzed, but their expression levels varied considerably. These genes can be effectively differentially expressed in different tissues. There were higher expression levels in the stem, and the expression of these genes tended to be upregulated after IAA treatment. The expression of FtAux/IAAs suggests that these genes could be involved in the regulation of buckwheat growth and development. This study will pave the way for further functional verification of the Aux/IAA gene family in buckwheat.

## 4. Materials and Methods

### 4.1. Plant Material and Hormone Treatments

Tartary buckwheat (Fagopyrum tataricum) seeds were sterilized, rinsed with sterile water, and sown in an improved Hoagland recipe. Plants were grown under standard greenhouse conditions, and the conditions in the culture chamber rooms were set as follows: 14 h day/10 h night cycle, 25/20°C day/night temperatures, 80% relative humidity, and 250 mmol m-2 s-1 intense luminosity. The roots, stems, and leaves at the seeding period were collected for expression analysis of the tissue-specific buckwheat auxin response gene family. Seeds with the same growth were treated with 10 *μ*mol L-1 IAA for 24 h in Hoagland liquid medium. All tissues and organs were stored at -80°C for RNA extraction.

### 4.2. Identification of the Auxin Response Gene Family in Buckwheat

The Tartary buckwheat genome was downloaded from the Tartary Buckwheat Genome Project (TBGP; available online: http://www.mbkbase.org/Pinku1/). The FtAux/IAA gene family members were identified using a BLASTp search. The FtAux/IAA genes were searched using two BLASTp methods, and the maximum number of Aux/IAA genes was determined. First, all known Arabidopsis Aux/IAA genes were used to query the initial protein on the TBGP website, and the candidate genes were identified using a BLASTp search at a score value of ≥100 and e − value ≤ 1 × 10 − 10. Second, the HMM file corresponding to the Aux/IAA domain (PF02519) was downloaded from the Pfam protein family database (http://pfam.sanger.ac.uk/). The Aux/IAA genes were retrieved from the Tartary buckwheat genomic database using HMMER3.0. The default parameter cutoff was set to 0.01. The existence of the Aux/IAA core sequences was verified with the PFAM and SMART programs, and the HMMER results of all candidate genes that might contain the Aux/IAA domain were further verified. The sequence length, molecular weight, isoelectric point, and subcellular localization of the Aux/IAA proteins were determined using the ExPasy website (available online: http://web.expasy.org/protparam/) ([69, 70].

### 4.3. Chromosomal Distribution Analysis of Aux/IAA Family Genes

All FtAux/IAA genes were mapped to the chromosomes from the physical location information obtained from the Tartary buckwheat genomic database using Circos [71]. Multiple collinear scanning toolkits (MCScanX) were used to analyze gene duplication events using default parameters [72]. To reveal the synteny relationship of orthologous Aux/IAA genes between Tartary buckwheat and other species selected, the syntenic analysis maps were constructed using the Dual Systeny Plotter software (available online: https://github.com/CJ-Chen/TBtools) [73]. The substitution of nonsynonymous (Ka) and synonymous (Ks) for each repeated Aux/IAA gene was calculated using the KaKs_Calculator 2.0 [74].

### 4.4. Gene Structure and Motif Characterization of FtAux/IAA Genes

Multiple sequence alignments of FtAux/IAAs were performed using DNAMAN through the highly conserved domains [24], to explore the structure of FtAux/IAA genes using the default parameter Clustal W [70]. In addition, the structural differences between FtAux/IAA proteins were predicted by comparing several conserved motif sequences with MEME Suite [75]. Motifs were evaluated using the Gene Structure Display Server (GSDS; http://gsds.cbi.pku.edu.cn/) with the following parameters: the optimum motif width was 6–200, and the maximum number of motifs was 20 [76].

### 4.5. Analysis of Phylogenetic Relationships

Phylogenetic analysis of all complete FtAux/IAA protein sequences was performed using the MEGA 7 program by the NJ method [69]. The phylogenetic trees were divided into different groups according to the conserved domain, and a bootstrap test was carried out with 1000 iterations [77, 78]. The same methods were applied to analyze the evolutionary relationships between buckwheat and Arabidopsis. In addition, the evolutionary relationships between buckwheat and rice, maize, and sorghum were analyzed using MEGA 7.

### 4.6. RNA Isolation and qRT-PCR Analysis

Total RNA was extracted using a total RNA extraction kit (Sangon, Shanghai, China, SK1321), and genomic DNA was removed with RNase-free DNase I treatment [12]. The first cDNA strand was generated by reverse transcription using M-MLV (TakaRa, Dalian, China), according to the manufacturer's protocol.

The gene expression level of the housekeeping gene histone 3 (GenBank ID: HM628903) of Tartary buckwheat was used as the endogenous control [79]. The gene-specific primers are summarized in Table 2, and the qRT-PCR reactions were performed in a total volume of 20 *μ*L (2 *μ*L diluted cDNA, 1 *μ*L each forward and reverse primer, 10 *μ*L SYBR Premix Ex Taq, and 6 *μ*L ddH2O). The qPCR program was as follows: 95°C for 3 min, followed by 30 cycles of 95°C for 15 s, 60°C for 30 s, and 72°C for 20 s. Gene expression was calculated using the 2-∆∆c method [80], and the mean of three biological replicates indicated their relative expression levels.

## Figures and Tables

**Table 1:** Aux/IAA family in buckwheat.

Gene ID	Chromosome	CDS (bp)	Introns	No. of aa	pl	MW (kDa)
FtPinG0008442000.01.T01	Chr1	1005	5	334	8.07	36303.04
FtPinG0008443000.01.T01	Chr1	2250	13	749	5.4	83729.44
FtPinG0000387700.01.T01	Chr1	1809	14	602	6.03	67736.99
FtPinG0004315700.01.T01	Chr1	678	4	225	6.06	24894.23
FtPinG0005029300.01.T01	Chr2	744	4	247	7.52	26767.1
FtPinG0000809900.01.T01	Chr2	1071	5	356	6.77	38851.25
FtPinG0000807700.01.T01	Chr2	2613	13	870	5.4	96264.2
FtPinG0006568700.01.T01	Chr3	591	2	196	6.63	21774.63
FtPinG0001961200.01.T01	Chr3	558	1	185	6.38	20844.57
FtPinG0007273100.01.T01	Chr3	798	3	265	8.42	29805.87
FtPinG0005142100.01.T01	Chr3	573	2	190	8.29	21405.48
FtPinG0005142700.01.T01	Chr3	621	4	206	5.44	22602.56
FtPinG0004530400.01.T01	Chr3	738	3	245	7.69	27386.98
FtPinG0005535200.01.T01	Chr4	615	1	204	5.98	23337.24
FtPinG0005745300.01.T01	Chr5	540	2	179	5.37	20362.13
FtPinG0007581000.01.T01	Chr6	693	2	230	8.23	25518.44
FtPinG0007581100.01.T01	Chr6	603	1	200	6.81	22823.65
FtPinG0001971700.01.T01	Chr6	2673	13	890	5.66	99377.01
FtPinG0002984800.01.T01	Chr6	915	4	160	7.66	33443.35
FtPinG0002846500.01.T01	Chr7	585	3	194	9.15	21554.57
FtPinG0007414500.01.T01	Chr7	696	4	231	6.62	25370.15
FtPinG0007414000.01.T01	Chr7	543	2	180	6.75	20280.1
FtPinG0007012600.01.T01	Chr7	552	1	183	5.58	20986.88
FtPinG0009157200.01.T01	Chr7	702	4	233	6.2	25555.18
FtPinG0009368700.01.T01	Chr8	1077	5	358	8.4	38717.69

The information listed in Table 1 was obtained from Tartary Buckwheat Genome Project. CDS: coding sequence; aa: amino acids; pl: isoelectric point; MW: molecular weight.

**Table 2:** Primer sequences of FtAux/IAA genes for qRT-PCR.

Name	Primer (5′- >3′)
FtAux/IAA 01	ATGGTGCTCCATATCTGCGG//CAATAGCGTCAGCGCCTTTC
FtAux/IAA 02	GAGCAAAGCGTCAGCAAACA//CTGGGTACCGTGAACTGCTT
FtAux/IAA 03	CCCTATTTCCTGCCAAGCCA//GGTCAACACCGAACAAACGG
FtAux/IAA 04	AGAAAAACGGCGATGTCCCT//CGAGTCCTATGGCTTCCGAC
FtAux/IAA 05	TGAGAACGATGTGGGAACCG//ACATCTTCTCCAAAGCCGCA
FtAux/IAA 06	GACTGGATGCTTGTGGGTGA//AATGGCGTCAGAGCCTTTCA
FtAux/IAA 07	ATTGCCCCAAGTAGGAAGCC//CCACGTGTTGTCGTGCAAAT
FtAux/IAA 08	GCTGTCCAAGAAGAACCCGA//CCATCCCACAATCTGTGCCT
FtAux/IAA 09	CGGGTTAATGGATCCGGGTT//ACGAACATCTCCCACGGAAC
FtAux/IAA 10	CGCAGCCTCCAAATCAATCG//AGACGCGCAACCTCTTTACA
FtAux/IAA 11	GGCCTCCAGTTTGCTCGTAT//CGAACGCTTTCGGTTCTTCC
FtAux/IAA 12	AGACAGAGCTCACTCTCGGT//GGCGACCAGAGAGGTTCAAA
FtAux/IAA 13	GCCGGTGAACTCATTCCGTA//AGCCGCTTTACGGTCGATAG
FtAux/IAA 14	CCAACCGACGACCACAAGTA//TATAGGATTGAACCGGCGGC
FtAux/IAA 15	TTCAATGGGGTCAACCTCCG//ACGAGCATCCAATCTCCGTC
FtAux/IAA 16	GGCCACCAGTGAGGTCATAC//ATCGCCGTCTTTGTCTTCGT
FtAux/IAA 17	GCACTTCTTCCGATGCAAGC//TGGTGGCCATCCAACAACTT
FtAux/IAA 18	CTCAGGGTCACAGTGAGCAG//AGTCGGACTAGCCCTTGGAT
FtAux/IAA 19	GAAGCTCCAAGCACCAATGC//TTTGAGCGGCAAGAAGACCT
FtAux/IAA 20	GTCACTGAACTCGCAAGGGA//CTCGCTTCCACATGCAAAGG
FtAux/IAA 21	AGAGGCTTCTCTGAGACCGT//TTCTCCGCGACCATTGACTC
FtAux/IAA 22	ACAACGTTGATGCCTCCGAA//ATAAGGTGCTCCGTCCATGC
FtAux/IAA 23	AAAAGACCCGAGAGCGATCC//CCCACGGAACATCTCCTACG
FtAux/IAA 24	GCCGTCCAAAAGAGTTGCAG//GACCAACATCCAATCCCCGT
FtAux/IAA 25	TTAAGGCTTGGACTGCCTGG//ATGGCGTCGGAACCTTTCAT

## Data Availability

The data used to support the findings of this study are available from the corresponding author upon request.
